# Dimethyl Sulfoxide Decreases Levels of Oxylipin Diols in Mouse Liver

**DOI:** 10.3389/fphar.2019.00580

**Published:** 2019-05-29

**Authors:** Poonamjot Deol, Jun Yang, Christophe Morisseau, Bruce D. Hammock, Frances M. Sladek

**Affiliations:** ^1^Department of Molecular, Cell and Systems Biology, University of California, Riverside, Riverside, CA, United States; ^2^Department of Entomology and Nematology and UCD Comprehensive Cancer Center, University of California, Davis, Davis, CA, United States

**Keywords:** DMSO, antioxidant, inflammation, pain, arthritis, obesity, oxylipins, diols

## Abstract

Dimethylsulfoxide (DMSO) is widely used as a solvent and cryopreservative in laboratories and considered to have many beneficial health effects in humans. Oxylipins are a class of biologically active metabolites of polyunsaturated fatty acids (PUFAs) that have been linked to a number of diseases. In this study, we investigated the effect of DMSO on oxylipin levels in mouse liver. Liver tissue from male mice (C57Bl6/N) that were either untreated or injected with 1% DMSO at 18 weeks of age was analyzed for oxylipin levels using ultrahigh performance liquid chromatography tandem mass spectrometry (UPLC-MS/MS). A decrease in oxylipin diols from linoleic acid (LA, C18:2n6), alpha-linolenic acid (ALA, C18:3n3) and docosahexeanoic acid (DHA, C22:6n3) was observed 2 h after injection with DMSO. In contrast, DMSO had no effect on the epoxide precursors or other oxylipins including those derived from arachidonic acid (C20:4n6) or eicosapentaenoic acid (EPA, C20:5n3). It also did not significantly affect the diol:epoxide ratio, suggesting a pathway distinct from, and potentially complementary to, soluble epoxide hydrolase inhibitors (sEHI). Since oxylipins have been associated with a wide array of pathological conditions, from arthritis pain to obesity, our results suggest one potential mechanism underlying the apparent beneficial health effects of DMSO. They also indicate that caution should be used in the interpretation of results using DMSO as a vehicle in animal experiments.

## Introduction

Dimethyl sulfoxide [DMSO, (CH_3_)_2_SO] is a polar aprotic compound with a high affinity for water (Brayton, 1986). It is commonly used as a solvent in biological experiments because it has low toxicity, can solubilize both polar and non-polar substances and can readily penetrate hydrophobic barriers such as the plasma membrane. These properties make it an ideal vehicle for both *in vivo* and *in vitro* experiments, especially for pharmacologic compounds that act on an intracellular level (Brayton, 1986).

Dimethyl sulfoxide has been reported to have therapeutic effects on a number of ailments including bacterial infections (Guo et al., 2016), dermatologic conditions (Lishner et al., 1985), chronic prostatitis (Shirley et al., 1978), gastrointestinal disorders (Salim, 1991, 1992a,b), pulmonary fibrosis and amyloidosis (Pepin and Langner, 1985; Iwasaki et al., 1994) arthritis (Elisia et al., 2016) and pain (Kingery, 1997; Kelava et al., 2011; Kumar et al., 2011; Rawls et al., 2017). Hepatoprotective effects of DMSO under various conditions of liver injury or hepatotoxicity have also been well documented (Siegers, 1978; Park et al., 1988; Achudume, 1991; Lind et al., 2000; Sahin et al., 2004). Although the physiological and pharmacological mechanisms underlying the beneficial health effects of DMSO are not fully known, they have been proposed to include its ability to increase blood flow to organs, decrease recruitment and activation of inflammatory cells and act as an antioxidant and free radical scavenger (Brayton, 1986; Beilke et al., 1987; Massion et al., 1996).

**FIGURE 1:**
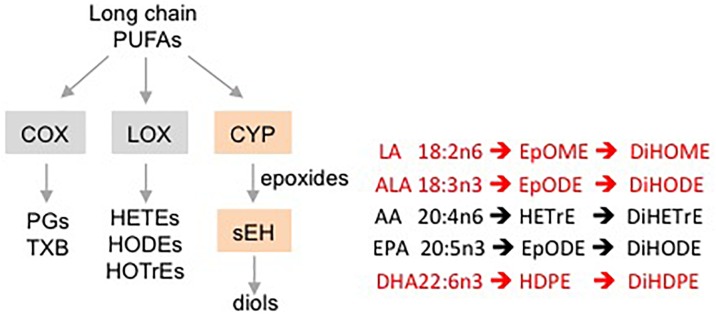
Schematic showing different pathways for metabolizing PUFAs to oxylipins. Examples of oxylipins generated by each of the three pathways metabolizing long chain PUFAs. Shaded boxes, enzymes. Pathways affected (orange, red text) or not affected (gray, black text) by DMSO in this study.

Oxylipins are biologically active, oxidized metabolites of long chain polyunsaturated fatty acids (PUFAs) that are generated by three different pathways – COX, LOX and CYP/sEH (Yang et al., 2009) (Figure 1). The third pathway consists of a two-step reaction involving the action of cytochrome P450s (CYPs) and soluble epoxide hydrolase (sEH) enzymes. This pathway first produces oxylipin epoxides and then diols from linoleic acid (LA, C18:2 n-6), alpha-linolenic acid (ALA, C18:3 n-3), arachidonic acid (AA, C20:4 n-6), eicosapentaenoic acid (EPA, C20:5 n-3) and docosahexaenoic acid (DHA, C22:6 n-3) (Moghaddam et al., 1997; Zeldin, 2001; Levick et al., 2007). Increased accumulation of oxylipin diols has been correlated with the pathogenesis of a number of pathological conditions including obesity, diabetes, depression, pain and cardiovascular disease (Gouveia-Figueira et al., 2015; Caligiuri et al., 2017; Deol et al., 2017; Hennebelle et al., 2017). Compounds that inhibit the formation of these lipid mediators, such as inhibitors of sEH (sEHI), have been shown to have therapeutic potential (Imig and Hammock, 2009; Morisseau et al., 2010; Wagner et al., 2017).

Dimethyl sulfoxide has been shown to attenuate the accumulation of lipids in the liver as well as free fatty acid-induced cellular lipotoxicity (Song et al., 2012). However, to our knowledge, the effect of DMSO on the hepatic levels of oxygenated fatty acid metabolites such as oxylipins has not been studied. Here, we investigate the effect of a single intraperitoneal injection of DMSO on the levels of approximately 60 oxylipin species in mouse liver. Our results show that DMSO lowers the levels of certain oxylipins, all of which are diols generated by the metabolism of omega-3 and omega-6 fatty acids LA, ALA and DHA.

## Materials and Methods

### Animals

Care and treatment of animals were in accordance with guidelines from and approved by the University of California, Riverside Institutional Animal Care and Use Committee (AUP #20140014). All mice had *ad libitum* access to regular vivarium chow (Purina Test Diet 5001, Newco Distributors, Rancho Cucamonga, CA) and water. At the end of the study, mice were sacrificed by CO_2_ inhalation followed by cervical dislocation, in accordance with stated NIH guidelines. C57BL/6N mice (Charles River Laboratories) were bred in-house and maintained on a 12h:12h light-dark cycle in a specific pathogen-free vivarium (SPF) with wood-chip bedding [PJ Murphy sani-chips 2.2 CF # 91100 (MFG 3-002)] and a cotton pad as an environmental stimulant. Pups were weaned at 3 weeks of age with three to four animals housed per cage.

### DMSO Treatment

Male mice (∼18 weeks old, *n* = 5 per group) were injected intraperitoneally with 200 μl of 1% DMSO (Sigma-Aldrich, catalog # D5879) and sacrificed 2 h later. About 200 mg of freshly excised liver tissue was rinsed in cold phosphate buffered saline (PBS), blotted with a Kimwipe and snap-frozen in liquid nitrogen for subsequent metabolomic analysis. Samples were also collected from a control group of age-matched mice that were not injected.

### Oxylipin Analysis

Non-esterified oxylipins were extracted by solid phase extraction from liver tissue homogenates (200 mg) and analyzed by ultrahigh performance liquid chromatography tandem mass spectrometry (UPLC-MS/MS) (Agilent 1200SL-AB Sciex 4000 QTrap) as described previously (Matyash et al., 2008; Yang et al., 2009; Deol et al., 2017). Analyst software v.1.4.2 was used to quantify peaks according to corresponding standard curves with their corresponding internal standards. Hepatic oxylipin concentrations are presented as pmol/gm tissue.

### Statistical Analysis

Data are presented as mean ± standard error of mean (SEM). Statistical significance is defined as *P* ≤ 0.05 using Student’s *t-*test.

## Results

**FIGURE 2:**
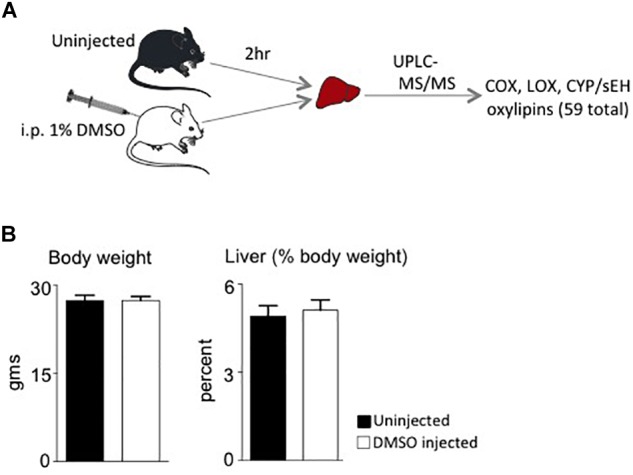
Study design and phenotypic data. **(A)** Workflow for the study showing the two cohorts of mice used and analyses performed. i.p., intraperitoneal. **(B)** Average body weights and liver weight as percent of body weight of male C57/BL6N mice at time of sacrifice (*N* = 5 per group).

**FIGURE 3:**
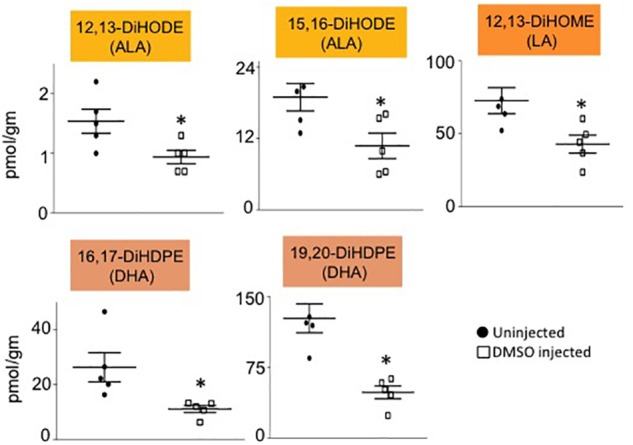
Liver oxylipin diol levels are decreased by DMSO. Absolute levels of diols that are significantly decreased in liver of mice injected with 1% DMSO (□) compared to uninjected controls (●). The fatty acid from which the oxylipin was derived is shown in parentheses. *N* = 5 mice per group. Data are presented as ±SEM. ^∗^Significantly different from uninjected control, *P* ≤ 0.05.

Male mice (∼18 weeks old) were injected with 1% DMSO and sacrificed 2 h later. Livers were removed and analyzed for oxylipins in the COX, LOX and CYP/sEH pathways (Figure 1, 2A). The 2-h time point was chosen to examine the short-term effects of DMSO and avoid potentially confounding factors that might be introduced by effects on gene expression. Not unexpectedly, the body weight and liver-to-body weight ratio at harvest did not differ between the control and injected groups (Figure 2B). Of the 59 oxylipin species analyzed, the DMSO-injected mice showed significantly altered levels of five species, all diols and all of which were decreased: 12,13-DiHODE, 15,16-DiHODE, 12,13-DiHOME, 16,17-DiHDPE and 19,20-DiHDPE (Figure 3). In addition, levels of two diols – 14,15-DiHETrE and 13,14-DiHDPE – were also lower in the DMSO group, although the decrease did not reach statistical significance (Supplementary Table 1).

**FIGURE 4:**
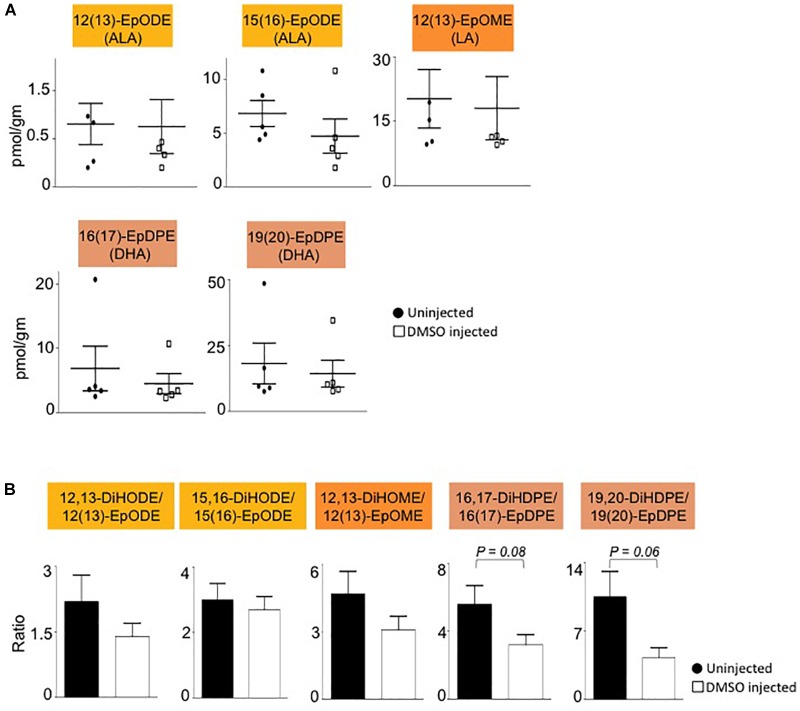
Liver epoxide levels and diol:epoxide ratios are not affected by DMSO. **(A)** Absolute levels of parent epoxides of diols (shown in Figure 3) in liver of mice injected with 1% DMSO (□) compared to uninjected controls (●). The fatty acid from which the oxylipin was derived is shown in parentheses. **(B)** Ratio of diol:epoxide as a measure of soluble epoxide hydrolase (sEH) activity. Color coding for parental fatty acid is same as in panel **(A)** and Figure 3. *N* = 5 mice per group. Data are presented as ±SEM. Significance is defined as *P* ≤ 0.05.

Interestingly, all of the oxylipins decreased by DMSO were in the CYP/sEH pathway and generated by hydrolysis of epoxides of LA, ALA and DHA. In contrast, levels of the epoxide precursors of these diols were not impacted by the DMSO treatment (Figure 4A). Diol:epoxide ratios, which are a reflection of sEH activity, were also not significantly different between DMSO and control, although the ratio for the DHA metabolites (DiHDPE:EpDPE) was trending toward significance (Figure 4B).

## Discussion

**FIGURE 5:**
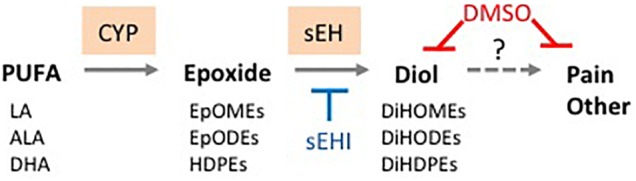
Proposed basis for therapeutic effects of DMSO. DMSO directly reduces oxylipin diol levels in tissues and thus helps mitigate pain and other symptoms associated with diol accumulation. Solid line, known reaction; dashed line, proposed causal effect. sEHI, soluble epoxide hydrolase inhibitor. See text for details.

Dimethyl sulfoxide is widely used to treat numerous ailments although the underlying mechanisms remain obscure. Our results show that a single injection of DMSO can cause an immediate and pronounced decrease in oxylipin diols generated from certain omega-3 and omega-6 PUFAs (LA, ALA and DHA) by the CYP/sEH pathway in mouse liver. It was proposed early on that one potential mechanism responsible for the physiological effects of DMSO was its ability to inhibit or activate various enzymes by reversibly altering their configuration (Rammler and Zaffaroni, 1967). DMSO has subsequently been shown to have a stabilizing effect on the RNA transcript levels of CYP enzymes in rat liver hepatocytes (Su and Waxman, 2004), and varying effects on CYP enzymatic activity depending on concentration, substrate and tissue or cell fraction (Chauret et al., 1998; Hickman et al., 1998; Li et al., 2010). At very high concentrations (28% *v/v*) DMSO has been shown to interact with the iron center of a bacterial cytochrome P450 enzyme (Kuper et al., 2012). We did not observe an alteration in the level of the precursor epoxides, suggesting that DMSO is not acting on the CYP enzymes in our system. Similarly, the diol:epoxide ratio was not significantly altered, suggesting that sEH activity was not altered. Interestingly, oxylipin epoxide and diol levels of two other PUFAs, AA and EPA, were not affected by DMSO. Combined with the relatively short time period needed to observe these effects (2 h), these results suggest that DMSO acts directly, and selectively, on LA, ALA and DHA oxylipin diols (Figure 5). It remains to be determined whether chronic DMSO treatment would show a similar selective effect.

Since the decrease in hepatic oxylipin levels in our experiments does not appear to be due to a decrease in enzyme action, this suggests that, at least in the short term, DMSO is either decreasing the stability or somehow preventing the accumulation of these compounds in the liver. DMSO has been reported to have antioxidant and free-radical scavenging properties (Sanmartín-Suárez et al., 2011; Kabeya et al., 2013) Thus, it is possible that DMSO may be acting as a scavenger for these oxidized metabolites, converting them into products that are not present in our oxylipin panel. Indeed, it has been reported that DMSO, when used as a vehicle, enhances the anti-inflammatory effects of rosemary and ginger (Justo et al., 2015). While the authors attributed this increase to better absorption and distribution of the compounds due to DMSO, it is possible that DMSO itself could have acted as an antioxidant, as has been shown previously (Alemón-Medina et al., 2008; Jia et al., 2010). These observations, along with the current results, indicate that caution should be employed when using DMSO as a vehicle to study the pharmacological efficacy of compounds with antioxidant potential. A direct, non-enzymatic effect of DMSO also suggests that it may have a similar effect in other tissues, and hence a broad applicability to numerous pathologies.

There are two other potential explanations for the reduced levels of the oxylipin diols. The first is that DMSO affects the level of the substrates, in this case LA, ALA and DHA. However, these fatty acids are essential (or in the case of DHA, conditionally essential), meaning that they must be derived from the diet. Consequently, the body has robust mechanisms to maintain their levels (Tove and Smith, 1960; Lin and Conner, 1990; Lin et al., 1993; Calder, 2016) making it unlikely that within 2 h of injecting DMSO there would be a large decrease in the steady state levels of these essential fatty acids. Furthermore, if the levels of the parental fatty acids were decreased, one would also expect to see decreased levels of the epoxides, which is not the case. The second possibility is that the diol levels decreased not because of the DMSO but because of the stress involved with the injection. However, we did not observe such effects in mock-injected mice in previous studies (Yang et al., 2009, 2013) and it is not likely that only certain oxylipins would be so significantly changed in a general stress response.

Oxylipin diols generated from omega-6 and omega-3 fatty acids have been associated with a number of pathologies including obesity, diabetes, and inflammatory and cardiovascular diseases (Kalupahana et al., 2011; Grapov et al., 2012; Tourdot et al., 2014; Deol et al., 2017). Thus, it is not surprising that limiting the production of diols with sEH inhibitors is emerging as an important therapeutic approach in disease management (Liu et al., 2012; Bettaieb et al., 2013; Wagner et al., 2017). Decreasing oxylipin diol levels by DMSO could be used as a treatment complementary to sEHI: while inhibition of sEH would help prevent the formation of new diols, DMSO would eliminate pre-existing diols that may have accumulated prior to sEHI treatment (Figure 5). For example, DMSO has been shown to mitigate inflammation in arthritis (Elisia et al., 2016), a disease associated with elevated levels of oxylipins (He et al., 2015; de Visser et al., 2018; Valdes et al., 2018). In one of these studies, however, a decrease in LA-derived diols was suggested to be causal for arthritis (He et al., 2015), indicating that additional investigation is needed (Figure 5).

Another example where DMSO could play a unique therapeutic role is in reducing extreme obesity. We have shown previously that all five of the oxylipin diols decreased by DMSO in this study – 12,13-DiHODE, 15,16-DiHODE, 12,13-DiHOME, 16,17-DiHDPE and 19,20-DiHDPE – correlate positively with soybean oil-induced obesity in mice (Deol et al., 2017). Soybean oil is by far the most commonly used cooking oil in the United States and is used ubiquitously in processed foods and restaurants (Blasbalg et al., 2011; Ash, 2012). While avoiding excess soybean oil in the diet is obviously preferable to taking any sort of medication, it is intriguing to speculate that in cases of intractable obesity, a compound such as DMSO that decreases elevated levels of diols might have a therapeutic effect. For such a treatment to work, however, the DMSO would need to have more than a transient effect on diol levels. Preliminary data from our lab suggest that this might be the case for at least one of the diols (not shown).

In summary, the results reported here provide new insights into the potential health effects of DMSO, and heightens our awareness of potential complications when using it as a solvent for therapeutic compounds.

## Ethics Statement

Care and treatment of animals were in accordance with guidelines from and approved by the University of California, Riverside Institutional Animal Care and Use Committee (AUP #20140014). All mice had *ad libitum* access to regular vivarium chow (Purina Test Diet 5001, Newco Distributors, Rancho Cucamonga, CA) and water. At the end of the study, mice were sacrificed by CO_2_ inhalation followed by cervical dislocation, in accordance with stated NIH guidelines.

## Author Contributions

PD and FS conceived and designed the experiments. FS and BH supervised the study. PD and JY performed the experiments. PD, CM, BH, and FS analyzed and interpreted the results. PD and FS wrote the manuscript with input from the other authors.

## Conflict of Interest Statement

The authors declare that the research was conducted in the absence of any commercial or financial relationships that could be construed as a potential conflict of interest.
